# Integrating phylogeographic patterns of microsatellite and mtDNA divergence to infer the evolutionary history of chamois (genus *Rupicapra*)

**DOI:** 10.1186/1471-2148-10-222

**Published:** 2010-07-22

**Authors:** Fernando Rodríguez, Trinidad Pérez, Sabine E Hammer, Jesús Albornoz, Ana Domínguez

**Affiliations:** 1 Departamento de Biología Funcional, Universidad de Oviedo, Genética, Julián Clavería 6, 33071 Oviedo, Spain; 2 Institute of Immunology, Department of Pathobiology, University of Veterinary Medicine Vienna, Veterinaerplatz 1, A-1210 Vienna, Austria

## Abstract

**Background:**

The chamois, distributed over most of the medium to high altitude mountain ranges of southern Eurasia, provides an excellent model for exploring the effects of historical and evolutionary events on diversification. Populations have been grouped into two species, *Rupicapra pyrenaica *from southwestern Europe and *R. rupicapra *from eastern Europe. However, a previous study of cytochrome b revealed that the two proposed species were non-monophyletic. The reconstruction of phylogenetic relationships between animal species often depends on the markers studied. To further elucidate the evolutionary history of chamois, we extended earlier studies by analysing DNA sequences of four mitochondrial regions (ND1, 12S, tRNApro and Control Region) and microsatellites (20 loci) to include all subspecies and cover its entire distribution range.

**Results:**

We found discordant microsatellite (μsat) and mitochondrial (mt) DNA phylogenies. Mitochondrial phylogenies form three clades, West, Central and East (mtW, mtC and mtE), at variance with taxonomic classification. Our divergence age estimates indicate an initial separation into branches mtW-mtC and mtE 1.7 million years ago (mya), in the late Pliocene-early Pleistocene, quickly followed by the split of clades mtW and mtC. Clade mtW contains haplotypes from the Iberian peninsula and the western Alps, Clade mtC includes haplotypes from the Apennines and the Massif of Chartreuse and Clade mtE comprises populations to the east of the Alps. Divergence among populations within these three major clades is recent (< 0.5 mya). New microsatellite multilocus genotypes added to previously published data revealed differences between every pair of subspecies, forming three well defined groups (μsatW, μsatC and μsatE) also with a strong geographic signature. Grouping does not correspond with the mitochondrial lineages but is closer to morphology and taxonomic classification. Recent drastic reductions in population size can be noted for the subspecies *ornata *as an extremely low diversity.

**Conclusions:**

The phylogeographic patterns for mtDNA and microsatellites suggest an evolutionary history with limited range contractions and expansions during the Quaternary period and reflect a major effect of the Alpine barrier on west-east differentiation. The contrasting phylogenies for mtDNA and microsatellites indicate events of hybridization among highly divergent lineages in the central area of distribution. Our study points to the importance of reticulate evolution, with periods of isolation and reduction of population size followed by expansions and hybridizations, in the diversification at the level of close species or subspecies.

## Background

Any group of organisms has a single true pedigree that extends back through time as an unbroken chain of parent-offspring genetic transmission but not all genes trickle through this pedigree in identical fashion [1]. Phylogenetic relationships within and between animal species often depend on the markers studied, as different genes might have different modes of transmission and different histories [2-4]. In addition, hybridization can result in discordant phylogenies between markers. Increasing evidence points to a contribution of reticulate evolution to the speciation process [5,6]. In this context, information on the phylogenies of different markers for closely related species and subspecies is important to the study of the processes underlying speciation [7].

**Figure 1:**
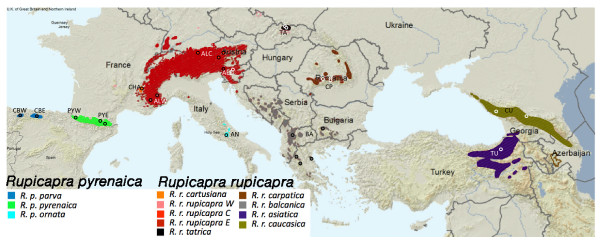
**Geographic distribution of the subspecies of the genus Rupicapra**. Sampling sites are indicated by circles and labelled with a letter code. The map was modified from the distribution map on the IUCN Red List [54].

The chamois (*Rupicapra *spp.) provides an excellent model for exploring the effect of historical and evolutionary events on diversification. It is distributed over most of the medium to high altitude mountain ranges of southern Eurasia (Figure 1). The Quaternary glacial ages probably had a major effect on the phylogeography and evolution of the genus *Rupicapra*, as it did on other animals in Eurasia [8-11]. There are diverse opinions concerning the phylogenetic relationships between fossil and living forms of Rupicaprini. The Rupicaprini seem to have originated in Asia during the Miocene period [11]; the fossil genus *Procamptoceras *appeared in Europe in the Villafranchian period (more than 2 million years ago [mya]) and together with *Rupicapra *is believed to belong to a phyletic lineage that had already separated from the ancestral Rupicaprini [12]. The sudden appearance of *Rupicapra *fossils in Europe during the middle Pleistocene age has been interpreted as resulting from immigration from the east during a cold climatic phase [11]. At present, chamois populations are classified into two species *R. pyrenaica *and *R. rupicapra *[13] on the basis of morphological and behavioural characters: *Rupicapra pyrenaica *(with the subspecies *parva*, *pyrenaica *and *ornata*) from southwestern Europe and *R. rupicapra *(with the subspecies *cartusiana*, *rupicapra*, *tatrica*, *carpatica*, *balcanica*, *asiatica *and *caucasica*) from northeastern Europe [14]. Analysis of genetic variation in a limited number of subspecies for allozyme loci [15], minisatellites [16], RFLPs of mitochondrial DNA [17] and the major histocompatibility complex [18,19] showed a considerably higher divergence between populations of the two proposed species than between populations within the same species. Microsatellite analysis of 8 of the 10 proposed subspecies showed a clear differentiation between every pair of populations and clearly separated two groups corresponding to the two proposed species of chamois [20]. The geographic distribution of separated mtDNA clades allows the study of historical demographic and dispersal events and the differentiation between mtDNA sequences can be used to date the separation among phylogenetic groups. The study of a fragment of cytochrome b (*cytb*) of 349 bp revealed that the two proposed species were non-monophyletic [21]. Three *cytb *lineages were identified: Clade West in the Iberian peninsula and western Alps, Clade Central in the Apennines and the Massif of Chartreuse and Clade East in populations to the east of the Alps. Clades West and Central are represented in both species, while Clade East is restricted to *R. rupicapra*. The divergence between the main clades has been estimated around 1.5-3 mya [21-25] but this cannot be directly assumed to be the divergence time between species. The study of microsatellites [20] has shown a correlation between genetic and geographic distance between populations, denoting a genetic flow among contiguous populations.

To further elucidate the processes leading to the diversification of the genus *Rupicapra*, we studied a larger sequence dataset including several mtDNA fragments and nuclear markers, which has been recommended to increase the performance of phylogenetic studies [26,27]. Earlier work was extended by analyzing DNA sequences of four mitochondrial regions [NADH Dehydrogenase subunit 1(ND1), 12S ribosomal RNA gene (12S), tRNAproline (tRNApro) and the control region (CR), total 1356 bp] and microsatellites (20 loci) to include all subspecies of chamois and to cover its entire distribution range. Here we include the subspecies *cartusiana *and *asiatica*, both missing from previous studies, as well as additional samples of *ornata *and *tatrica *(previously only represented by 1 and 2 individuals respectively). Comparison of the geographic distribution of mitochondrial and nuclear markers allow us to follow the pattern of hybridization between highly differentiated lineages in the context of the climatic oscillations of the Pleistocene age.

## Results

### Mitochondrial DNA phylogeography

We have amplified and sequenced fragments of ND1, 12S, tRNApro and CR from 152 individuals. These sequences were concatenated with a fragment of *cytb *obtained in a previous study [21]. The combined dataset contains 1708 nucleotides (1646 nt, indels excluded), 742 of them corresponding to coding sequences. The alignment resulted in 79 haplotypes defined by 219 variable sites of which 64 corresponded to coding regions. The overall number of mutations was 223, of which 15 corresponded to non-synonymous substitutions in coding regions. Mitochondrial DNA diversity was high (Table 1) with on average one distinct haplotype over 1.9 individuals (152/79). The Alpine chamois (*R. rupicapra rupicapra*) as a whole showed very high values of diversity, both haplotypic (95.07%) and nucleotidic (2.80%). In particular, nucleotide diversity was very high in the sample from Val di Susa in the western side of the Alps. On the other hand, diversity was extremely low for populations from the Massif of Chartreuse (*R. rupicapra cartusiana*) and from the Apennines (*R. pyrenaica ornata*).

**Table 1:** Estimates of diversity at mitochondrial sequences

		tRNApro			12S			ND1			CR			Combined		
subspecies	n	**n**_**h**_	% h	% π	**n**_**h**_	% h	% π	**n**_**h**_	% h	% π	**n**_**h**_	% h	% π	**n**_**h**_	% h	% π
*parva*	15	1	0	0	1	0	0	2	41.90 [11.32]	0.4265 [0.2969]	9	92.38 [4.40]	2.5936 [1.4034]	**9**	**92.38 [4.40]**	**0.8274 [0.4418]**
*pyrenaica*	26	1	0	0	1	0	0	4	64.31 [ 7.17]	0.2075 [0.1714]	12	90.77 [03.31]	1.7289 [0.9336]	**13**	**91.08 [3.39]**	**0.5156 [0.2745]**
*ornata*	12	1	0	0	1	0	0	1	0	0	1	0	0	**2**	**16.67 [13.43]**	**0.0101 [0.0164]**
*cartusiana*	8	1	0	0	1	0	0	1	0	0	3	46.43 [20.00]	0.1647 [0.1588]	**3**	**46.43 [20.00]**	**0.0412 [0.0397]**
*rupicapra W*	18	2	20.92 [11.63]	0.9507 [0.8735]	4	52.94 [11.70]	0.4829 [0.3174]	4	65.36 [9.82]	1.1176 [0.6472]	8	88.89 [4.16]	4.4847 [2.3334]	**9**	**90.85 [3.91]**	**1.7563 [0.9014]**
*rupicapra C*	20	1	0	0	2	10.00 [8.80]	0.0235 [0.0467]	3	19.47 [11.45]	0.0509 [0.0736]	8	78.42 [8.40]	1.7693 [0.9657]	**8**	**78.42 [8.40]**	**0.5043 [0.2724]**
*rupicapra E*	11	1	0	0	2	18.18 [14.36]	0.0428 [0.0673]	4	49.09 [17.54]	0.1758 [0.1622]	9	94.55 [6.59]	2.5772 [1.4359]	**9**	**94.55 [6.59]**	**0.7092 [0.3929]**
*tatrica*	10	1	0	0	1	0	0	1	0	0	3	37.78 [18.13]	0.5340 [0.3638]	**4**	**73.33 [10.05]**	**0.1661 [0.1086]**
*carpatica*	16	1	0	0	2	12.50 [10.64]	0.0294 [0.0532]	2	40.00 [11.35]	0.1018 [0.1115]	10	86.67 [7.93]	2.1015 [1.1477]	**11**	**87.50 [8.10]**	**0.6172 [0.3334]**
*balcanica*	9	1	0	0	3	72.22 [9.67]	0.4444 [0.3167]	2	50.00 [12.83]	0.2545 [0.2135]	6	88.89 [9.10]	4.3420 [2.4188]	**6**	**88.89 [9.10]**	**1.2623 [0.6995]**
*asiatica*	1	1	-	-	1	-	-	1	-	-	1	-	-	**1**	**-**	**-**
*caucasica*	6	1	0	0	1	0	0	3	73.33 [15.52]	0.2375 [0.2168]	4	80.00 [17.21]	1.9094 [1.1965]	**4**	**80.00 [17.21]**	**0.6156 [0.3792]**
**TOTAL**	152	3	60.26 [1.97]	2.2399 [1.5086]	10	67.56 [2.24]	1.1466 [0.6222]	21	89.26 [1.37]	2.7187 [1.3777]	74	97.99 [0.39]	8.4529 [4.0968]	**79**	**98.27 [0.35]**	**3.6169 [1.7379]**

n, number of individuals analysed; n_h_, number of haplotypes observed; h, haplotype diversity; π, nucleotide diversity. Standard deviations [SD] are shown in brackets.

**Figure 2:**
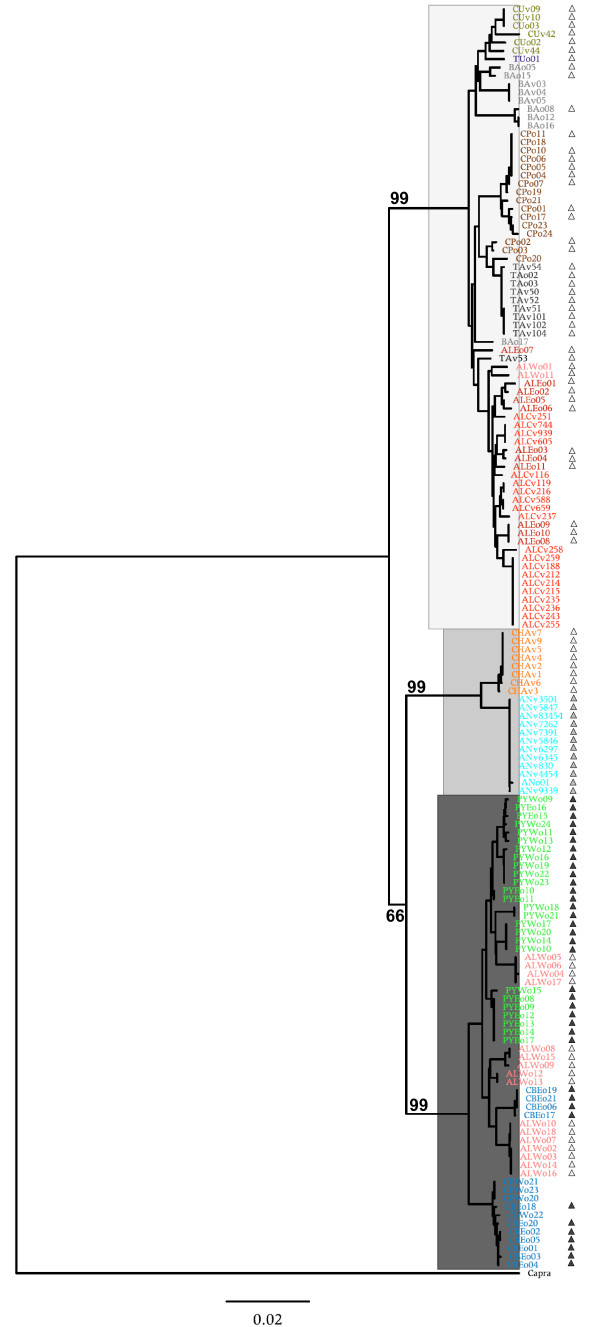
**Tree of 152 chamois based on the combined sequence**. Neighbor-Joining tree based on the number of substitutions per nucleotide under the model of Jukes-Cantor. Bootstrap support is shown at the nodes. Tip labels contain the unique individual identifier that includes the sampling site in the form of a capital-letter code (as depicted in Figure 1). Colours indicate the recognized subspecies as in Figure 1. Clade mtW, Clade mtC and Clade mtE indicate the three major mitochondrial lineages in black, grey and white. Coloured triangles in black, grey and white indicate the affiliation of individuals to microsatellite Clades μsatW, μsatC and μsatE.

**Figure 3:**
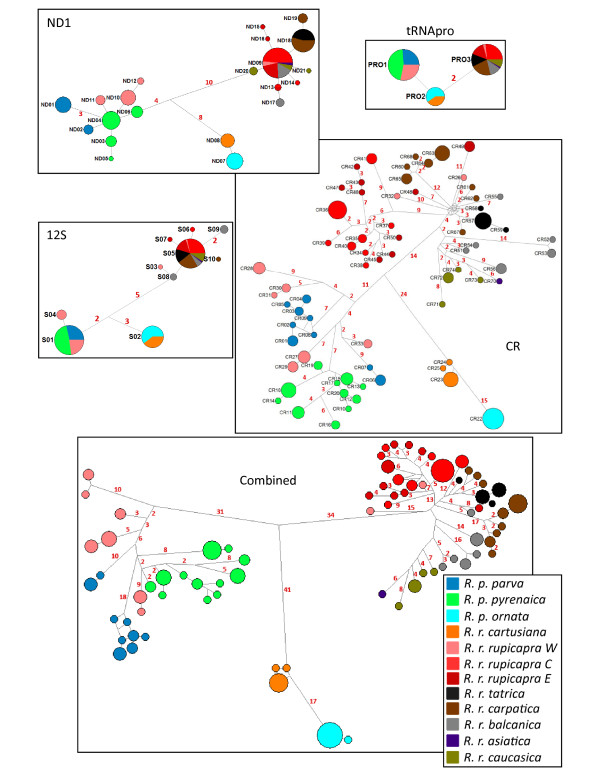
**Networks of mitochondrial haplotypes**. Median-joining networks for the mtDNA fragments of ND1, 12S, tRNA-pro, the Control Region and for the combined sequence (which in addition includes *cytb*). The size of pie areas corresponds to haplotypic frequencies and the proportion accounted for by the different subspecies is represented in different colours as in Figure 1. Branch lengths greater than 1 between haplotypes are indicated as a red number on the branches. Branch lengths are not scaled.

A simple Neighbor-Joining tree based on Jukes-Cantor distances between individuals (Figure 2) revealed three well supported major clades, although these do not concur with the taxonomy of chamois. The three clades, hereinafter named Clade mtWest (mtW), Clade mtCentral (mtC) and Clade mtEast (mtE), show a strong geographic signal. Clade mtW is present in individuals from the Iberian peninsula (*R. pyrenaica*) and the western Alps (*R. rupicapra*), Clade mtC in individuals from the Apennines (*R. pyrenaica*) and the Massif of Chartreuse (*R. rupicapra*) and Clade mtE in all individuals of populations from the central Alps to the Caucasus. Thus, the two species were mitochondrially non-monophyletic: *R. pyrenaica *contains the clades mtW and mtC and *R. rupicapra *the three mitochondrial clades. Networks computed using the four mitochondrial fragments (ND1, 12S, tRNApro and CR) separately (Figure 3) showed identical topologies as the combined analyses with the same three major clades, indicating that the four datasets contain solely mitochondrial fragments and no nuclear pseudogenes. Total network lengths are different for the four datasets, representing the different rates of nucleotide substitution among different segments. The ND1 fragment shows 21 haplotypes defined by 37 variable sites, the 12S fragment presents 10 haplotypes and 19 variable sites, the tRNApro 3 variable sites defining 3 haplotypes and the CR, 74 haplotypes and 132 variable sites (indels excluded) (The GenBank accession numbers for the different haplotypes are listed in Additional file 1). The haplotype network of the combined sequence is characterized by the three main clades. Every haplotype is limited to a single population and haplotypes within each clade are connected to haplotypes of the same population or of a nearby population. However, the haplotypes of the western Alps are connected to haplotypes of the Cantabrian Mounts and the Pyrenees but are quite differentiated from them. The haplotypes of Chartreuse belong to Clade mtC and occupy a more central position within the network than the haplotypes from the Apennines. Pairwise differences between populations in mean number of substitutions per nucleotide (Table 2) are all significant with the exception of the differences between individuals from the central and the eastern Alps, *rupicapra E *versus *caucasica *and *asiatica *versus *caucasica*.

**Table 2:** Pairwise differences between populations for nuclear microsatellites (F_ST_) above diagonal; and mtDNA (Net mean number of substitutions per site under Jukes-Cantor), below diagonal

	*parva*	*pyrenaica*	*ornata*	*cartusiana*	*rupicapraW*	*rupicapraC*	*rupicapraE*	*tatrica*	*carpatica*	*balcanica*	*asiatica*	*caucasica*
*parva*		14.31	51.43	42.51	33.31		35.65	48.42	45.05	36.41	40.10	42.27
*pyrenaica*	0.65		50.18	44.22	33.59		35.64	48.36	45.84	37.29	39.83	43.22
*ornata*	4.42	4.58		70.91	50.41		59.68	77.14	67.21	64.23	93.12	69.46
*cartusiana*	4.24	4.54	1.15		11.31		20.16	36.58	34.67	18.42	44.31	29.22
*rupicapraW*	0.52	0.32	4.13	4.01			6.35	24.99	20.38	11.18	17.73	13.85
*rupicapraC*	4.81	4.98	5.81	5.46	3.94							
*rupicapraE*	4.67	4.80	5.72	5.37	3.74	0.15		29.06	22.73	12.53	19.21	16.23
*tatrica*	4.59	4.89	5.42	5.11	3.85	1.11	0.77		25.55	21.81	47.36	38.35
*carpatica*	4.81	5.11	5.41	5.10	4.06	1.15	0.86	0.86		22.52	31.71	27.23
*balcanica*	4.17	4.46	5.02	4.87	3.43	0.91	0.85	0.98	0.68		16.38	20.43
*asiatica*	4.84	4.97	6.01	5.83	4.05	1.47	0.83	1.57	1.32	1.33		20.61
*caucasica*	4.42	4.72	5.51	5.40	3.71	1.20	0.34	1.05	1.00	0.98	0.51	

To investigate further the evolutionary history of *Rupicapra*, the 79 haplotypes of the combined dataset were aligned with sequences of *Capra hircus*, *Ovis aries *and *Bos taurus *and the phylogenetic relationships were investigated using Maximum Likelihood, Maximum Parsimony, Neighbor-Joining or Bayesian approaches under different models of nucleotide substitution. Following the Hierarchical Likelihood Ratio Test (hLRTs), as implemented in MODELTEST [28], the combined dataset of haplotypes with outgroups was found to fit a HKY + I + G model. The six parameters (nucleotide frequencies A:0.3588, C:0.2540, G:0.1331, T:2540; Ts/Tv ratio: 16.037; rate heterogeneity: 0.3464 and proportion of invariants: 0.4791) given by MODELTEST were used to obtain an ML tree with the program DNAML of the PHYLYP package [29]. The number of different possible evolutionary rates was set to five plus a class of invariant sites. The same model of nucleotide substitution was used for the construction of the Bayesian tree but the parameters were obtained by the program Beast itself. There were 196 parsimony-informative sites. Model-free Parsimony Analysis performed with MEGA [30] led to thirty equally parsimonious trees with a total length of 1150 steps. NJ analysis was performed by means of the simple model of Jukes-Cantor.

**Figure 4:**
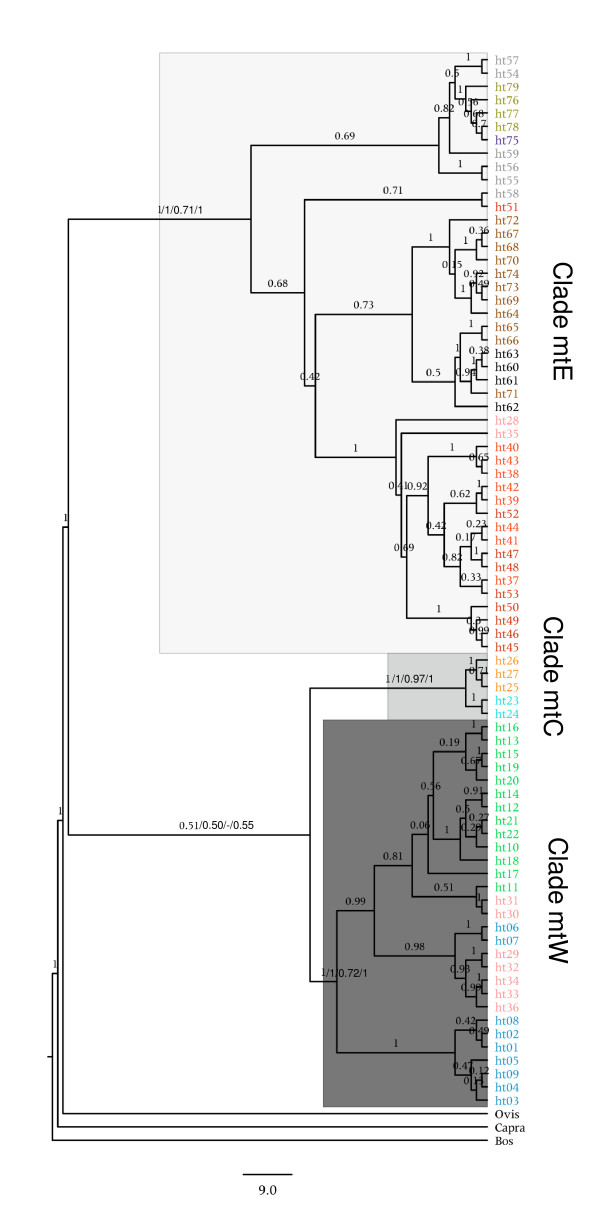
**Mitochondrial DNA phylogeny of chamois**. Phylogeny of chamois constructed by Bayesian analysis of the 79 haplotypes resulting from the combined sequences (1708 nt). Bayesian posterior probabilities are shown above each branch and, in addition, NJ, ML and MP bootstrap support indices are shown in the main interior branches.

The different methods of tree construction all led to topologies with three main well supported branches (Figure 4). In addition, Clade mtC divides into two well supported external branches representing the chamois from the subspecies *R. p. ornata *and *R. r. cartusiana*. The other two major clades, mtW and mtE, do not show consistent external nodes. Only an external node including several Cantabrian haplotypes is formed within Clade mtW and, similarly, only an external node of haplotypes from the Carpathians forms in Clade mtE. These groups must correspond to local lineage sorting rather than to long phylogenetic divergence.

The relationships between the three major clades or internal branches were found to vary depending on the method used for tree construction. Under ML, the split between Clades mtC and mtE is posterior to the split of Clade mtW. The topology obtained with Bayesian, MP and NJ methods (in Figure 4) was always poorly supported, suggesting that the divergence of these three main clades most probably happened in a radiation within a short period of time.

Using the divergence times of Bovidae, Caprinae and Capra-Ovis as calibrations, following Hernandez-Fernandez and Vrba [31], the divergence of Clades W-C and E was dated at 1.68 mya (95% confidence limits [CI]: 0.91-2.56), overlapping with confidence limits for the time of divergence between Clades W and C, which was calculated to be 1.37 mya (95% CI: 0.75-2.09). The subsequent divergences within these three main clades are considerably younger (< 0.5 mya), already in the middle Pleistocene.

### Microsatellite DNA phylogeography

The number of alleles per locus ranged from 2 to 23 with a mean of 9.20 (see Additional file 2). Observed heterozygosities were, in general, slightly lower than expected (Table 3) and the difference was significant in the subspecies *carpatica *and *balcanica*. Only one combination individual -locus failed to amplify, indicating that null alleles do not occur at high frequency. The test with Micro-Checker identified potential null alleles at frequencies higher than 0.2 in the subspecies *carpatica *(SR-CRSP-13, SR-CRSP-14) and *balcanica *(SR-CRSP-8, SR-CRSP-12, ETH225, INRA036). After the exclusion of the loci with potential null alleles, the observed heterozygosity was still lower than expected (*carpatica*, P = 0.0023; *balcanica*, P = 0.0061). Hence, the heterozygote deficit in these subspecies can be attributed to the Wahlund effect rather than to the presence of null alleles. The locus SR-CRSP-14 is identified by Micro-Checker as having potential null alleles at a frequency of 0.24 in *rupicapraW*. The fact that this population showed no general heterozygote deficit suggests that deviation is probably due to the presence of null alleles. Despite this finding, the effect in overall F-statistics and genetic distances would be limited and hence the locus was retained for analysis. The subspecies *tatrica *showed a diversity of 33%, in the lowest range of the values. The population of the Apennines showed an extremely low diversity of 3%. Six out of 12 individuals were homozygous for the 20 loci and only 3 loci presented more than one allele.

**Table 3:** Estimates of diversity for 20 nuclear microsatellites

Species	Subspecies	n		P	A	PA	Rs	%He	%Ho	
	*parva*	40	(11)	17	4.45	3	3.36	51.31	47.00	
*R. pyrenaica*	*pyrenaica*	41	(26)	17	5.20	10	3.70	51.66	48.29	
	*ornata*	12	(12)	3	1.15	1	1.11	3.15	3.33	
	*cartusiana*	8	(8)	15	2.90	2	2.81	42.00	43.75	
	*rupicapra W*	20	(18)	19	4.85	3	3.87	58.13	52.75	
	*rupicapra E*	11	(11)	19	4.20	0	3.77	55.37	53.18	
*R. rupicapra*	*tatrica*	10	(10)	15	2.45	0	2.25	33.39	32.50	
	*carpatica*	17	(10)	18	3.35	4	2.86	43.45	35.29	*
	*balcanica*	9	(3)	17	4.00	5	3.74	55.00	38.89	*
	*asiatica*	1	(1)	10	-	1	-	-	52.63	
	*caucasica*	10	(6)	15	3.80	6	3.36	42.55	40.50	

n, number of individuals analysed, in brackets individuals also typed for mtDNA; P, number of loci polymorphic; A, mean number of alleles; PA, number of private alleles; Rs: allelic richness (calculated based on a minimum sample size of 7 diploid individuals); Ho, observed heterozygosity; He, expected heterozygosity. *P < 0.001.

**Figure 5:**
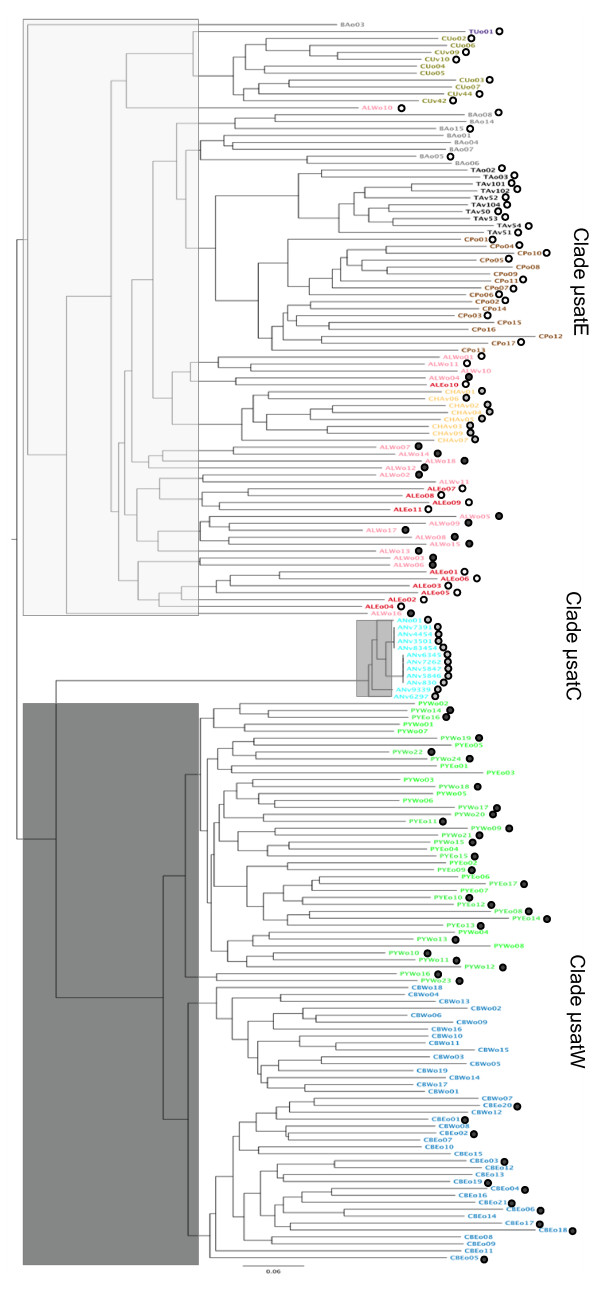
**Microsatellite phylogeny of chamois**. Neighbor-Joining tree based on allele-sharing distance from 20 microsatellite genotypes of 179 *Rupicapra *individuals. Tip labels contain the unique individual identifier that includes the sampling site in the form of a capital-letter code (as depicted in Figure 1). Colours indicate the recognized subspecies as in Figure 1. Clade μsatW, Clade μsatC and Clade μsatE indicate the three major nuclear lineages in black, grey and white. Coloured circles in black, grey and white indicate the affiliation of individuals to mitochondrial Clades mtW, mtC and mtE.

A Neighbor-Joining tree of 179 individuals (116 of them also included in the mitochondrial analysis) based on allele-sharing distance (Figure 5) shows two main clades corresponding to the Iberian chamois (Clade μsatW) and the Eastern chamois (Clade μsatE) and a third one (Clade μsatC) that groups the Apennine chamois, the subspecies *R. pyrenaica ornata*. Like the mitochondrial tree, the microsatellite-based phylogeny shows a strong geographic signal but at the centre of the distribution of *Rupicapra *the mitochondrial and the nuclear data are in apparent conflict. All eight individuals of *R. rupicapra cartusiana *formed with *R. pyrenaica ornata *the mitochondrial Clade mtC but group with alpine chamois *R. rupicapra rupicapra *for microsatellite markers. In contrast to mitochondrial data, the 18 individuals sampled from the west Italian Alps group with the nuclear Clade μsatE, while 16 of them belong to Clade mtW (see Figure 2). The microsatellite tree shows the clustering of individuals of the different subspecies even though there is not a clear-cut between them.

**Figure 6:**
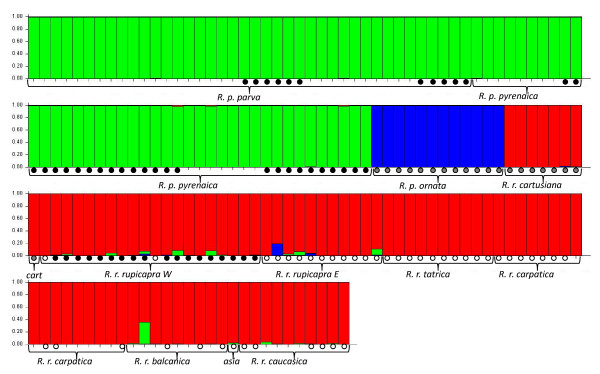
**Graphic representation of the STRUCTURE of microsatellite variation**. Each vertical bar represents one individual and its assignment proportion into one of the three clusters. Circles in black, grey and white, indicate the affiliation of individuals to mitochondrial Clades mtW, mtC and mtE.

Bayesian clustering of individuals with the software Structure using the method of Evanno et al. [32] yields a likely number of clusters of two. Nevertheless, inspection of the twenty replicate runs of Structure shows inconsistencies among replicates, with Clade μsatC as defined above clustered with μsatW in eight of the replicates and with μsatE in the remaining 12 replicates. Clustering of individuals with K = 3 (Figure 6) is more consistent among replicates and forms the same groups of individuals as the microsatellite tree in 17 replicates. In the other three, the grouping of the subspecies *parva*, *pyrenaica *and *ornata *varies: in two of them *pyrenaica *and *ornata *group together and *parva *forms a different group but in one replicate *parva *and *ornata *group together. Higher orders of structure (K = 7-9) yield clusters that tend to group individuals of the same or neighbour populations but the clusters present low consistency among replicates.

**Figure 7:**
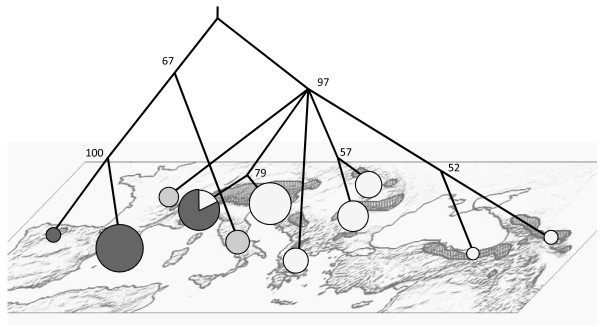
**Summary of geographic distribution of mitochondrial and nuclear variation**. A UPGMA consensus tree generated from Nei's Standard Genetic Distance for microsatellites is represented over the map. Values at nodes indicate bootstrap support. Pies on the map correspond to the three mitochondrial clades in different shades of grey, as in Figures 2 and 4. Pie areas correspond to mitochondrial clade frequencies.

Every pairwise comparison of genetic differentiation between populations (excluding the comparisons with *asiatica *represented only by one individual) differs significantly from zero (Table 2). A UPGMA consensus tree was generated from 1000 bootstrap replicates based on Nei's standard genetic distance. The population tree topology was represented over the geographic distribution of the genus (Figure 7) to highlight the geographic signature on the microsatellite variation.

## Discussion

Our phylogenetic analysis based on either mitochondrial or nuclear DNA variation gives results that are to a certain some extent discordant, even though both markers show a strong east/west phylogeographic signal that must be related to the distribution of lineages in space and time with recurrent periods of isolation and contact in contiguous areas of the species' range. The discordance between mitochondrial phylogeny and the taxonomic classification, based mostly on morphological characters, results in non-monophyly. The two species of *Rupicapra*,* R. pyrenaica *and *R. rupicapra*, are not reciprocally monophyletic for mtDNA; Clades mtW and mtC are represented in both, while Clade mtE is restricted to *R. rupicapra*. It is interesting that *R. rupicapra cartusiana *groups with *R. pyrenaica ornata *regarding the mtDNA, as both form the Clade mtC, but clusters with its conspecific group *R. rupicapra *on the basis of microsatellite variation. No signs of recent admixture can be noted in the individual nuclear genotypes. The population from the west Italian Alps (*R. r. rupicapra W*) is non-monophyletic with haplotypes belonging either to the Clade mtE (2 individuals) or to the Clade mtW (16 individuals). These phylogenetic relationships can be interpreted either as a consequence of old hybridization among differentiated lineages [21] or as a result of much more recent human-mediated translocations [25]. The different expectations of the two hypotheses are as follows: if translocation and hybridization were recent, within the last 150 years as suggested (24 generations assuming a generation time of 6.24 years following Gaillard [33]), a signature of the reintroduction should be visible both at the levels of nuclear and mitochondrial variation. As evident from the Structure analysis, the 16 individual alpine specimens of the Clade mtW belong to the nuclear Clade μsatE and there are no signs of admixture or recent hybridization. In addition, close inspection of the subsample of haplotypes of Clade mtW from alpine *R. r. rupicapra *individuals argues against a recent reintroduction because: 1) the haplotypic and nucleotidic diversities (0.883 and 0.00786) are high and similar to the values found, for example, in the Pyrenean chamois; 2) none of the seven haplotypes present in this subsample were recovered from the Pyrenees. Our Pyrenean sample is limited in number and the samples come only from two sampling locations (see Figure 1) but it is nevertheless remarkable that locations on both sides of the Pyrenees share haplotypes with each other but not with the sample from the Alps; 3) the genetic distance, in mean number of substitutions per nucleotide (following Jukes-Cantor), between the subsample of Clade mtW from the Alps and either the Cantabrian or the Pyrenean chamois (1.38 and 0.99) is comparable to the distance among the Cantabrian and Pyrenean populations themselves (0.65) and hence denotes a similar time of divergence. Thus we conclude that the haplotypes from Clade mtW present in the alpine *R. r. rupicapra *population result from ancient hybridization.

Overall, phylogeographic analysis of mtDNA and μsatDNA allows the definition of three groups of chamois that separate in an east-west pattern. The two types of markers gave incongruent results for individuals from the regions of contact between lineages, the Massif of Chartreuse and the western Alps. Hence, our results provide strong evidence for the effect of old migrations and hybridization between highly differentiated lineages on the current composition of populations in the central area of the distribution of chamois.

### Taxonomic implications

The currently accepted taxonomy of chamois recognizes two species: *R. pyrenaica*, which include chamois from the Iberian peninsula together with the chamois from the Appenines; and *R. rupicapra*, which includes all the other populations [13]. However, the taxonomy of the genus has been subject to continuous revisions during the twentieth century. In 1914, Camerano [34] distinguished the species *R. ornata *on the basis of skull and horn morphometrics. Subsequently Couturier and Dolan considered the ten populations of chamois as a single species [35], [40] but later work based on skull evaluations [36], electrophoretic data [15] and different coat pattern as well as several courtship behaviour patterns [37] suggested that treatment as two species is warranted. More recently, Crestanello et al. [25] suggested that *R. pyrenaica ornata *be re-elevated to species rank in accordance with the high divergence between the mtC Clade and the other two. However, these authors did not take into account that *R. rupicapra cartusiana *also belongs to the Clade mtC.

The mitochondrial DNA data provide information about phylogeny that is frequently used to diagnose species using the phylogenetic species concept (PSC). Evolutionary Significant Units (ESUs), essentially equivalent to species under the PSC [38], have been defined as populations of individuals reciprocally monophyletic for mtDNA alleles and differing significantly in the frequency of alleles at nuclear loci [39]. According to this criterion, mitochondrial phylogeny implies that a single species of chamois (*Rupicapra rupicapra*) should be recognized, as by Couturier [35] and Dolan [40]. However, experiments on species delimitation that are based on markers from a single uniparentally inherited genome must be treated with caution, given that such markers are preferentially introgressed across species boundaries [4,41]. Multilocus assignment methods have been proposed to have considerably more power [4]. The microsatellite analysis clearly separates three groups: two corresponding to the two recognized species plus a third group for individuals from the Apennines, that are closer to the Iberian chamois than to the other populations. This finding can be related to the classification proposed by Camerano [34], who accorded the rank of species to the population from the Apennines (*R. pyrenaica ornata*). Morphological differentiation between *ornata *and *pyrenaica *has also been shown by Scala and Lovari [42], even though differences between the Iberian and Apennine group and all other *Rupicapra rupicapra *spp. are much greater [36]. Thus, microsatellite differentiation seems to be more closely related to morphological variation than does work with mtDNA. Mitochondrial phylogenies are frequently discordant with taxonomy, and hence with morphological differentiation [43-47]. In the case of chamois, we have shown that the introgression of mtDNA into the Eastern chamois corresponds to ancient hybridizations. Our conclusion is consistent with the observation of intermediate phenotypes between *R. rupicapra *and *R. pyrenaica *in *R. r. cartusiana *[36], implying that the consequences of hybridization were not limited to introgression of mtDNA from local animals to the invading species. From the perspective of the biological species concept, only one species should be considered if there is widespread hybridization among the nominal species. Further information on the extent of past hybridization in the central area of the distribution is therefore required to define the taxonomy of *Rupicapra*.

Overall, the genus *Rupicapra *exhibits levels of diversity comparable to those found in other genera of wild Artiodactyla in Europe [48-52]. Microsatellite markers show a differentiation among populations of the ten currently recognized subspecies, though differences are not always clear-cut. Most populations of chamois show intermediate levels of diversity either for microsatellites or for mtDNA. Even though the limited number and non-random distribution of samples precludes a detailed comparison of intra-population variability, some of the data are remarkable. The subspecies *rupicapra*, represented by many thousands of individuals, has very high levels of diversity, both for mitochondrial and for microsatellite markers. Previous studies have documented the existence of genetic fragmentation at this geographic scale [25,53]. The high level of diversity at the mtDNA level in the sample from the western Alps can be attributed to ancient hybridization of very different lineages, as we have discussed. The subspecies *ornata *and *cartusiana*, which were classified as vulnerable and endangered by the Caprinae Specialist Group [54], show very low diversities at the level of mitochondrial DNA: 0.01% and 0.04%, respectively. These low levels of mitochondrial diversity can be related to reduced female population sizes in the past due to geographic isolation. With regard to diversity for microsatellites, the subspecies *cartusiana *shows a moderate level of 42%, similar to other subspecies, while the subspecies *ornata *presents the extremely low value of 3%. Six out of 12 individuals are homozygous for the 20 loci and only three loci present more than one allele. This level of diversity is lower than the lowest values reported for several bottlenecked mammalian populations: H _e _= 0.43 for a bighorn sheep population founded from 12 individuals (Forbes 1995), H _e _= 0.25 for the brown bear subpopulation isolated in the east Cantabrian mountains [55], H _e _= 0.13 for a Mexican grey wolf population founded with fewer than ten individuals [56] or H _e _= 0.13 for the alpine Ibex population of the Alpi Marittime-Mercantour, which was reintroduced between 1920 and 1933 with an effective number of founders possibly lower than ten animals [57]. The diversity of the subspecies *ornata *for microsatellites is possibly the lowest value reported in the literature for a population of non-selfing diploid organisms. This is a reflection of the recent past of the Apennines population, with two extreme bottlenecks in the last century. The subspecies *ornata *nearly became extinct early in the 20 ^th ^century and in the late 1940s [37] and recovered to 800 animals by 2003 [58].

### Inferences on the evolutionary history of chamois

**Figure 8:**
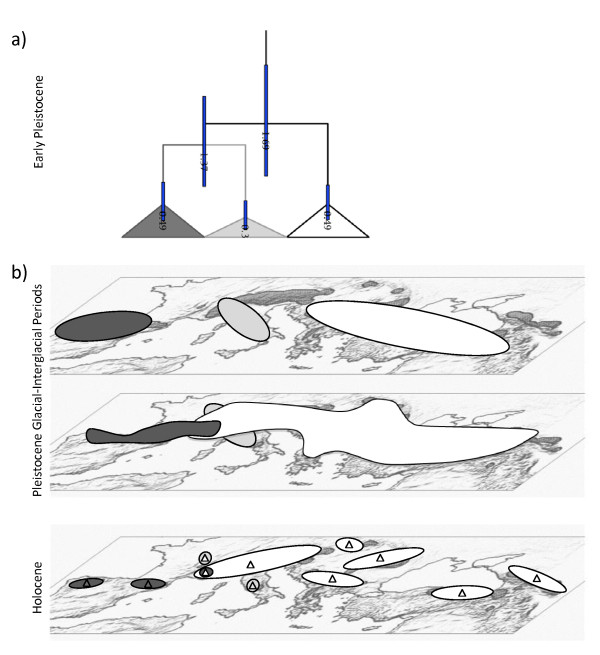
**Divergence age estimates and hypothetical evolutionary history of chamois, along the Quaternary**. a) Collapsed tree with divergence age estimates resulting from BEAST analysis. The mean age estimate for each node is given in million years, with 95% credibility intervals indicated by the blue bars. The Clades mtW, mtC and mtE are represented in colours black, grey and white. b) Hypothetical evolutionary history of chamois along the Quaternary. The affiliation to Clades μsatW, μsatC and μsatE of extant populations of chamois is represented by a triangles coloured in black, grey and white.

The distribution of variation, both of mtDNA and of microsatellites, shows a clear geographic signature with a west-east differentiation. Present day mtDNA clades show an east-west distribution along medium to high-altitude mountain ranges of southern Europe and the near east: each clade of mtDNA forms a patch occupying a delimited geographic area, even though Clade mtC is split into two with both in the central area of distribution. For microsatellites, genetic and geographic distances have been shown to correlate [20], consistent with gene flow among populations. For both types of markers the barrier of the Alps is a factor that disrupts the distribution of genetic variation. The somewhat contrasting pictures offered by the two types of markers can be related to their different modes of evolution. Microsatellite markers narrate the phylogenetic history of tens of thousands of years while mitochondrial markers shed light on the deeper phylogenetic history [59]. In addition, both markers, especially mtDNA, provide information about phylogeographic events such as migration and hybridization of populations. The largely concordant geographic distribution of both old and new genetic variation in chamois implies that differentiation occurred without major migrations since the establishment in Europe of the three extant mitochondrial lineages (Figure 8).

The earliest *Rupicapra *fossils stem from the middle Pleistocene and have been found in France, together with *Hemitragus *and *Ovis*, and a few remains from the Rissian age have been found in the Pyrenees, the Italian Alps, the Apennines and Hungary [11]. Masini and Lovari [11] have suggested that the chamois, or its direct ancestor, may have reached the European region as a late immigrant during the Early or Middle Pleistocene, probably from southwest Asia. The phylogenetic analysis of 1708 nucleotides, including five mitochondrial genes, concurs with previous studies [21,25]. It shows an initial split of the *pyrenaica *(mtW-mtC) and *rupicapra *(mtE) lineages 1.7 mya, based on the molecular clock. Clade mtC started to diverge from mtW very soon after, 1.4 mya. The divergence time estimates between the three lineages have overlapping confidence intervals and place the radiation back to the Plio-Pleistocene before the beginning of the strong climatic oscillations of the Quaternary. The contrast between this dating and that obtained from microsatellites [20] can be attributed to the well known effect of homoplasy of microsatellites, which leads to underestimation of separation times for long diverging populations [60]. Thus, molecular mitochondrial data place the age of modern chamois lineages before their first occurrence in the fossil record. In addition, an older mitochondrial lineage fossilized in the nucleus as a pseudogene has been identified in both species currently recognized [24]. Its translocation to the nucleus was close to the radiation of present day clades, suggesting the existence of older chamois precursors in Europe of a lineage that did not survive until the present.

The geographic patterns of mtDNA and microsatellite variation suggest that the three major mitochondrial clades differentiated "in situ" with moderate migrations after their initial radiation. In the middle Pleistocene, chamois occurred in the geographic area they currently occupy [11]. There have probably been multiple phases of isolation and hybridization between contiguous populations, most likely caused by expansions to lower altitudes during Pleistocene glacial periods and contractions to high altitudes during interglacial periods. The presence of chamois at high altitudes in the Swiss Alps during the Riss-Würm interglacial period and its wider and continuous distribution on low altitude sites during the Würm has been documented [11]. The ice sheets in the Alps and the Pyrenees during glacial maxima must have constituted barriers that greatly limited contacts between populations already showing a pattern of isolation by distance. Clade West was presumably isolated to the west of the Pyrenees, in the Iberian peninsula; Clade Central must correspond to the isolation of chamois, most probably between the Pyrenees and the Alps; and Clade East was probably isolated to the east of the Alps during the glacial maxima and presumably extended its distribution during interglacial periods. It is highly likely that Clade West recolonized the western Alps and there encountered the lineage from the east that occupied most of the Alps. The populations constituting Clade mtC were probably split by the expansion of the two main clades into the central region. This interpretation is consistent with the paleontological evidence for the presence of *R. rupicapra *spp. in Holocene deposits of the northern Apennines [11]. The subspecies *cartusiana*, which lives in the isolated mountain system of Chartreuse on the western edge of the French Alps, carries mitochondria from Clade mtC, while nuclear markers place it in the eastern group (μsatE), denoting hybridization. This observation is in accordance with the hypothesis of Lovari and Scala [36], who argue that hybridization might explain why *R. r. cartusiana *bears some phenotypes that are intermediate between *R. rupicapra *and *R. pyrenaica*. Parallel data were observed with regard to the population from Val di Susa in the western Alps, where most individuals carry the mitochondrial Clade mtW together with nuclear markers of the Clade μsatE, denoting hybridization among lineages in the contact zone. Finally, the warm climate of the Holocene definitively isolated the populations, which were restricted to the tops of the different mountain ranges.

Our data concur with other studies on comparative phylogeography in Europe [9,10] in explaining the divergence between lineages in the context of divergence among three main areas and of the effect of the Alpine barrier in population differentiation. The historical events of population range contractions and expansions due to climatic oscillations may have eliminated haplotypes present in glacial areas and led to hybridizations between other lineages. Our findings are consistent with a scenario of diversification of the genus *Rupicapra *without major migrations since the time of radiation of present-day clades but involving periodic isolation of populations and subsequent range overlap, most probably triggered by climatic changes, and hybridization.

## Conclusions

The mitochondrial phylogeny shows three main lineages that originated in a close period at the Early Pleistocene. There is a first split of the Clades mtW-mtC from mtE (dated 1.7 mya), soon followed (1.4 mya) by the split of the left branch into Clades mtC and mtW. The two nominal species of chamois *R. pyrenaica *and *R. rupicapra *are not monophyletic for mtDNA. Microsatellite genotypes form three well defined groups that do not exactly match the mitochondrial lineages but are closer to morphology and taxonomic classification. Based on all these findings, *Rupicapra *populations are subdivided into three main groups: the Iberian populations, the Apennine population and northeastern populations. The geographic signature in the distribution of variability suggests that differentiation occurred without major migrations. The phylogeographic patterns suggest an evolutionary history with range contractions and expansions related to climatic oscillations during the Quaternary period and reflect a major effect of the Alpine barrier on west-east differentiation. The contrasting phylogenies for mtDNA and microsatellites for populations of Chartreuse and the western Alps indicate events of range overlap and hybridization among highly divergent lineages in the central area of the distribution. In addition, the extremely reduced variability of some subspecies shows the potential importance of lineage sorting in the composition of present-day populations.

Our study points to the importance of reticulate evolution, with periods of isolation and reduction of population size followed by expansion and hybridization, in the diversification of close species.

## Methods

### Mitochondrial DNA and microsatellites - Sampling and DNA Extraction

Samples of the 10 recognized subspecies of chamois were collected from 1992 until the present, covering the distribution range of the genus *Rupicapra *(see Figure 1). A total of 215 samples were analyzed either for microsatellites and mtDNA (116 samples) or for just one type of marker (63 samples for microsatellites only and 36 samples only for mitochondrial markers) (see Additional file 3). From the 179 samples analyzed for microsatellites, 142 had been previously typed [20]. The 37 new samples included individuals from populations lacking (*cartusiana *and *asiatica*) or poorly represented (*ornata*, *tatrica*, *balcanica *and *caucasica*) in the previous study. For large populations, where hunting is allowed, samples were either of muscle or skin preserved in 96% ethanol by gamekeepers, or teeth from skulls sent to taxidermists. For protected populations, samples were obtained from animals found dead; tissues, as well as their conservation method, were diverse (hair, bone, salted skin and muscle in ethanol) and were sent by biologists.

Due to the different origin and type of the material included in this study, different methods of DNA isolation were used. DNA from bones or teeth was extracted by a method modified from Catanneo et al. [61] as described [20]. For soft tissue samples, DNA was extracted either with the phenol/chloroform method [62] using Chelex, following Estoup et al. [63] or using the 'DNeasy Tissue kit' (Qiagen, Hilden, Germany). Finally, 56 of the 215 samples were collected and the DNA extracted in the laboratory of Vienna (Austria) following the protocol described in the Genetic Analysis Manual (LI-COR, Inc. 1999). The lysed sample was subjected to a standard phenol/chloroform extraction and DNA precipitation procedure [62]. DNA extracted in Vienna was sent to Oviedo (Spain) for analysis.

### Mitochondrial DNA Sequencing Analysis

Four mitochondrial sequences corresponding to the tRNApro gene, parts of NADH-1 (ND1), 12S rRNA (12S) genes and the Control Region (CR) were sequenced. A fragment between 488 bp and 547 bp, including 6 bp to the right of the tRNA-thr that was discarded, the tRNA-pro (66 bp) and the left hypervariable region (HVR-I) (416-475 bp) of the CR, was amplified with the primers CRa F (5'-AGGAGAACAACTAACCTCCC-3') and CR R (5GGTTTCACGCGGCATGG'-3') designed from the sequences of *R. rupicapra *in the GenBank (AM279274 and AM279275). Primers for amplification of ND1 were designed from the sequences of *R. pyrenaica *(GenBank DQ236338) and *R. rupicapra *(GenBank DQ236339) [64]. A fragment of 444 bp, including 51 bp of the ARNt-leu, was generated with the primers ND1F (5'-GTGGCAGAGCCCGGTAATTG- 3') and ND1R (5'-TGTGCTACTGCTCGTAAGGC-3'). For the 12S rRNA gene, the primers 12SbF (5'-ACAAAATTATTCGCCAGAGTACT-3') and 12SR (5'-TCCAGTATGCTTACCTTGTTACG'-3') were designed from the sequence of *R. rupicapra *(GenBank AM158314) and produced a fragment of 471 bp. PCRs conditions for all amplifications were identical. Reactions were performed in a final volume of 20 μl containing 2 μl (≈ 40-70 ng) DNA, 0.5 mM of each primer, 1× PCR Buffer, 200 mM of each dNTP, 2.5 mM MgCl _2 _and 0.5 U of Taq DNA polymerase (Qiagen, Hilden, Germany). Amplification was carried out in PE GeneAmp PCR 9700 thermal cycler (Applied Biosystems, Foster City, CA) with an initial step of 3 min at 94°C, 30-35 cycles of 15 s at 94°C, 30 s at 62°C and 30 s at 72°C, followed by 10 min at 72°C. PCR products were electrophoresed along with size standards in 2% agarose gel in 1× Tris-borate-EDTA and visualized by UV. The PCR-amplified products were purified with the Exo-SAP-IT kit (USB Corporation, Cleveland, OH) and sequencing reactions performed with the previous designed primers and the BigDye Terminator v3.1 Cycle Sequencing Kit (Applied Biosystems). Sequencing products were purified with isopropanol precipitation and sequenced in an ABI 310 Genetic Analyzer (Applied Biosystems). The raw sequence data were analyzed using the ABI Prism DNA Sequencer Analysis software v3.4.1.

### Mitochondrial DNA - Phylogenetic Reconstruction

The mitochondrial sequences were aligned using the multiple alignment program of BioEdit [65] and manually checked and edited. All generated haplotypes of the four studied fragments were submitted to NCBI GenBank (accession numbers GU951809-GU951916, see Additional file 1). In addition, the four datasets plus a fragment of *cytb *previously sequenced in the same individuals (accession numbers EU836150-EU836161 and EU836163-EU836168, see Additional file 1)[21] were combined to produce a final alignment of 1708 nucleotides (1646 nt, indels excluded). Sequences were analyzed separately for the four data sets and for the combined dataset with the MEGA4 software package [30] and DnaSP 4.0 [66]. A Neighbor-Joining tree based on the number of substitutions per site under the Jukes-Cantor model was constructed from the combined sequences of the 152 *Rupicapra *individuals. All positions containing gaps were eliminated (complete deletion option in MEGA). Haplotype diversity (h) and nucleotide diversity (π) were estimated for each subspecies. The evolutionary genetic distance between pairs of subspecies was quantified with MEGA as the net average number of substitutions per site. Analyses were conducted using the Jukes-Cantor model of nucleotide substitution and the Standard Errors obtained by a bootstrap procedure (1000 replicates). Significance of these inter-group distances was tested with a Z-test performed with EXCEL and applying the Bonferroni correction [67].

The evolutionary relationships between the haplotypes, of the four markers separately or the combined sequence, were analyzed by a Median-Joining network [68] constructed with NETWORK 4.2 (Fluxus Technology Ltd.). This method differs from traditional ones by allowing extant haplotypes to occupy internal nodes. The parameter ε was set to zero (default) to obtain a sparse spanning network. For the CR and the combined datasets the Median-Joinig network was enhanced by first running the Reduced-Median network (with the Reduction Threshold Parameter r set to the default value of 2) to simplify the outcome. Phylogenetic relationships were further analyzed for the dataset of haplotypes of the combined sequence aligned with sequences of *Capra hircus *(AF533441), *Ovis aries *(NC_001941) and *Bos taurus *(NC_001567) as outgroups. Neighbor-Joining (NJ), Maximum Parsimony (MP), Maximum-Likelihood (ML) or Bayesian approaches were used under different models of nucleotide substitution. We elected not to use sophisticated models of nucleotide substitution for analyzing phylogenies because differences in genetic estimates of distances are low when closely related sequences are studied. In addition, statistical prediction based on a model with many parameters is subject to larger error variance [69]. A Neighbor-Joining (NJ) tree of haplotypes based on Jukes-Cantor distance was constructed with MEGA. The reliability of the nodes was assessed by 1000 bootstrap replicates [70]. The topology of the tree was further investigated by model free Maximum Parsimony (MP) as implemented in MEGA. The MP tree was obtained using the Close-Neighbor-Interchange algorithm with search level 3 in which the initial trees were obtained with the random addition of sequences (10 replicates). The MP consensus tree was inferred from 1000 bootstrap replicates with MEGA. The Maximum Likelihood (ML) tree was obtained using the DNAML program within the PHYLIP package [29], after determining the optimal substitution model from the hierarchical Likelihood Ratio Test (hLRTs) implemented in MODELTEST 3.7 [28]. To assess the reliability of the nodes, 1000 bootstrap replicates were obtained with the program SEQBOOT within the PHYLIP [29] and analysed with the program DNAML under the multiple dataset option. The consensus tree and the bootstrap support were obtained with TreeAnnotator of the software package BEAST [71].

Bayesian analysis was conducted using the Monte Carlo Markov chains (MCMC) method implemented in BEAST [71]. A relaxed lognormal model of lineage variation and a coalescent prior with constant size was assumed given that the alignments contain multiple intraspecific sequences [72]. The model of nucleotide substitution was HKI + G + I with the empirical nucleotide sequences and a gamma distribution of site heterogeneity with 5 categories of substitution rates plus invariant sites as priors. Two replicates were run for 25 million generations with tree and parameter sampling every 1,000 generations. A burn-in of 10% was used and the convergence of all parameters assessed using the software TRACER (within the BEAST package). Subsequently, the sampling distributions of two independent replicates were combined using the software LogCombiner and the resulting samples summarized using the software TreeAnnotator and visualized with FigTree [73]. Divergence times were estimated with BEAST, which employs a relaxed molecular clock approach. As calibration we used the divergence times of Bovidae (mean 25.8 mya, standard deviation [SD] 0.6 mya), Caprinae (mean 14.1 mya, SD 1.1) and Capra-Ovis (11.5 mya, SD 0.9) following Hernández-Fernández and Vrba [31] as a normal distribution prior. We placed monophyly constrains on the group Caprinae and on the group *Rupicapra*.

### Microsatellite Markers and Multiplex PCR

The twenty polymorphic microsatellite loci described previously [20,74] were analyzed. The amplification conditions were as described but fluorescent labelled primers were used and several markers were co-amplified and/or co-loaded in the same well for analysis. Five multiplex reactions were developed to amplify 12 of the loci (ETH-10 + ETH225; INRA005 + INRA023; SR-CRSP-6 + SR-CRSP-8; SR-CRSP-1 + SR-CRSP-3 + SR-CRSP-14; SR-CRSP-9 + SR-CRSP-12 + SR-CRSP-15). The remaining 8 loci (INRA003; INRA011; INRA036; INRA063; SR-CRSP-4; SR-CRSP-5; SR-CRSP-11; SR-CRSP-13) were amplified independently. Amplification was carried out using the PE GeneAmp PCR 9700 (Applied Biosystems). PCR products were checked in a 2% agarose gel and the product diluted up to 100-fold depending on the signal intensity. One microlitre of the dilution was added to a 12 ml mix of formamide and ROX 400HD (12:0.2) and loaded on an automatic sequencer ABI310 (Applied Biosystems). Several PCR reactions were co-loaded for analysis: (INRA036 + [ETH10 + ETH225], INRA003 + [INRA005 + INRA23], INRA11 + INRA63) and the remaining seven PCR reactions were loaded independently. To obtain the complete profile of each individual sample 13 PCR reactions and 10 runs were needed. Microsatellite patterns were examined both visually and using GENESCAN ANALYSIS 3.1 and GENOTYPER 2.5 software (Applied Biosystems).

### Microsatellites - Statistical Analyses

Multilocus individual genotypes were arranged in a matrix of 20 loci per 179 individuals (142 typed in a previous study and 37 individuals added in this study). Multilocus genotypes were complete for all but the only individual from the subspecies *asiatica*, for which the locus SR-CRSP-4 could not be amplified.

Descriptive statistics analysis was performed with GENEPOP [75,76] and MSA [77]. In each population, every locus was tested for departure from Hardy-Weinberg (HW) by the "exact HW test" [76]. The algorithm used to estimate the exact P-value was a Markov-chain method with the default values recommended by the authors. Global tests across loci for each population were constructed using Fisher's method. The Bonferroni procedure was applied to correct the significance level for multiple comparisons [67].

Genotyping errors and null alleles were evaluated with Micro-Checker [78] for each population. This method, along with all other methods for detecting null alleles, assumes that deviations from HW do not result from other causes, such as the Wahlund effect. We estimated the frequency of potential null alleles with Micro-Checker following the method of Brookfield, indicated when failures in the amplification of just a single locus (which could signify a null homozygote) are not observed. Frequencies of null alleles lower than 0.2 are not expected to cause significant problems in analyses [79]; thus we only considered loci exceeding this value to be potentially problematic.

The allele-sharing distance between every pair of individuals [80] was calculated using MSA [77] and a Neighbor-Joining tree was constructed from the resulting distance matrix using the program NEIGHBOR of the PHYLIP package [29]. The tree was rooted in the midpoint. Population structure was detected with the software STRUCTURE 2.1 [81] (without prior population information), which uses a Markov chain Monte Carlo (MCMC) algorithm to define the most likely genetic clusters on the basis of multilocus genotype data. We used different values of K, from one to ten, and ran STRUCTURE 20 times for 100,000 steps after a burn-in period of 50,000 steps. The correct value of K was estimated following Evanno [32] and by visual inspection of the replicates. Population differentiation was investigated with F _ST _[76] using MSA and significance was tested by 10,000 bootstraps and applying the Bonferroni procedure. Several studies have tested the performance of different genetic distance measures in resolving the evolutionary relations of closely related populations or species from microsatellite data [82,83]. The results have shown that Nei's standard distance, *Ds *[84] performs well. We calculated *Ds *with the software MSA [77]. Bootstrapping over loci for *Ds *was achieved with MSA. These multiple data sets (1000 replicates) were used to construct UPGMA trees with the NEIGHBOR program from PHYLIP 3.5c [29]. The 50% majority-rule consensus tree was generated with the CONSENSE program in PHYLIP 3.5c. Tree diagrams were obtained with FigTree [73].

## Authors' contributions

FR ran much of the sequence data collection and undertook analyses and interpretation. TP ran the bulk of the microsatellite data collection and undertook analyses and interpretation. SEH and JA carried out aspects of the molecular labwork and manuscript composition. AD conceived and coordinated the study, analysed mtDNA and microsatellite data and wrote the paper. All authors read and approved the final manuscript.

## Supplementary Material

Additional file 1 **GenBank accession numbers of mitochondrial sequences**. Click here for file

Additional file 2 **Estimates of diversity for each nuclear microsatellite/population pair**. n, number of individuals analysed;  A, number of alleles, in brackets PA, number of private alleles; Range, allelic size range; Rs, allelic richness (calculated based on a minimum sample size of 7 diploid individuals). Ho, observed heterozygosity; He, expected heterozygosity. Values departing from Hardy-Weinberg, after Bonferroni correction, are shown in bold (p < 0.05 in both cases). Click here for file

Additional file 3 **List of samples**. List of samples analysed in this study along with locality, year of sampling and haplotype designation for the mtDNA fragments. Samples genotyped for microsatellites are marked with an asterisk. Click here for file
